# Congenital Zika Virus Infection with Normal Neurodevelopmental Outcome, Brazil

**DOI:** 10.3201/eid2411.180883

**Published:** 2018-11

**Authors:** Alessandra Lemos de Carvalho, Carlos Brites, Tânia Barreto Taguchi, Suely Fernandes Pinho, Gúbio Campos, Rita Lucena

**Affiliations:** SARAH Network of Rehabilitation Hospitals, Salvador, Brazil (A.L. de Carvalho, T.B. Taguchi, S.F. Pinho);; Federal University of Bahia, Salvador (C. Brites, G. Campos, R. Lucena)

**Keywords:** Zika virus, neurodevelopment, outcome, viruses, Brazil, congenital Zika syndrome

## Abstract

We describe a case of a 20-month-old girl with probable congenital Zika virus infection and normal neurodevelopment, despite microcephaly and abnormal neuroimaging. This case raises questions about early prognostic markers and draws attention to the need for investigation in suspected Zika cases, even if the child’s early neurodevelopment is normal.

Zika virus is a mosquitoborne RNA virus (genus *Flavivirus*, family Flaviviridae) that was first isolated in 1947 from monkeys in the Zika Forest in Uganda (*1*). In November 2015, there was an outbreak of congenital microcephaly in the northeast states of Brazil (*2*). Further confirmation of this syndrome’s relationship with Zika virus infection during pregnancy was then possible (*3*). Congenital Zika syndrome has been recognized as a new clinical entity (*4**,**5*). Most published case series focus on the picture of severely affected infants (*6**,**7*). We describe a case of a child with probable congenital Zika virus infection whose neurodevelopment was normal, despite microcephaly and abnormal neuroimaging. The mother provided written informed consent for this report.

The patient, a girl, was born at 36 weeks’, 4 days’ gestation; Apgar scores were 8 at first minute and 9 at fifth minute. There was no family history of microcephaly, and the parents were phenotypically normal. The pregnancy occurred during Brazil’s Zika virus epidemic and the mother lived in Bahia, the state where the virus was first detected and one of the most affected areas. She was 27 years of age, in her third pregnancy, and had a history of rash at 12 weeks’ gestation, followed by fever, headache, arthralgia, and conjunctivitis. She recovered after 1 week, without a specific diagnosis. At 24 weeks’ gestation, a routine ultrasound exam detected microcephaly in the fetus. Testing for HIV, human T-lymphotropic virus, cytomegalovirus, toxoplasmosis, rubella, syphilis, and hepatitis B and C in the mother yielded negative results. She had no further complications except for high blood pressure detected 3 days before delivery, the discovery of which led to an elective cesarean section.

At birth, the infant’s weight was 2,496 g (−0.6 SD), length was 45 cm (−1.1 SD), and head circumference was 29.5 cm (−2.4 SD) (*8*). She had no neonatal complications and was breast-fed without difficulty. Test results were negative for chikungunya, dengue, rubella, toxoplasmosis, cytomegalovirus, parvovirus B19, and herpes virus I and II. However, serum testing (EuroImmun, Lubeck, Germany) showed positive results for Zika virus IgM. Results of biochemical analysis of cerebrospinal fluid, abdominal ultrasound, and neonatal metabolic screening were all normal, as were ophthalmologic and auditory evaluations. Transfontanellar ultrasound showed focal calcification in basal ganglia that was more pronounced in the right hemisphere. Results of cerebral computed tomography conducted during the neonatal period showed mild craniofacial disproportion, slightly decreased brain volume, and small calcifications in the right nucleocapsular area and around the left thalamus. 

In January 2016, at 6 months of age, the patient entered a neurorehabilitation program, during which she reached normal achievement of developmental milestones. Further investigation included a videoelectroencephalogram and auditory and visual evoked potentials; results were all normal. We diagnosed probable congenital Zika virus infection, considering the gestational history, congenital microcephaly, positive serologic test results, and neuroradiological findings, which were mild but consistent with Zika virus infection. A follow-up cerebral scan performed at 7 months of age showed mild calcifications at the right lenticular nucleus and posterior arm of the left internal capsule (Figure). 

**Figure:**
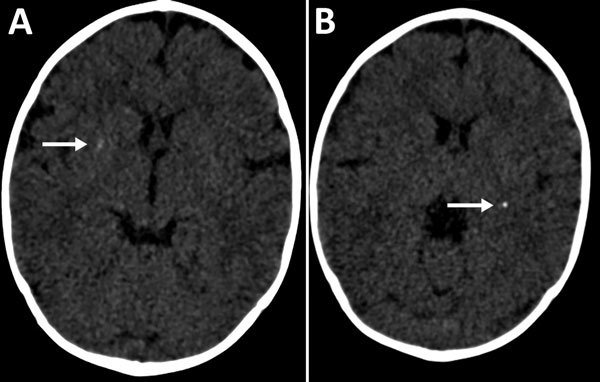
Cerebral computed tomography images of infant with probable congenital Zika virus infection at 7 months of age, Brazil. A) Mild calcification at the right lenticular nucleus (arrow); B) calcifications at the posterior arm of the left internal capsule (arrow).

The patient started to walk independently at 13 months of age, and her gait pattern did not show any abnormality. We performed a follow-up assessment at 20 months of age. Neurologic examination was normal, except for microcephaly (head circumference 42.5 cm, −3 SD) (*9*). Weight was within reference range (8.7 kg, between −1 and −2 SD), but length was below reference values (74 cm, between −2 and −3 SD). The child was otherwise healthy. We performed developmental evaluation using Bayley-III Scales of Infant Development (*10*). All composite scores were within average classification (Technical Appendix).

Neurodevelopment is a dynamic process that depends on the interaction between neurobiological and environmental factors. Children initially developing within the expected range for their age group may experience a slowdown as demands of neurodevelopment become more extensive. We cannot make inferences about long-term prognosis from the patient’s condition at 20 months of age. Developmental evolution should be determined prospectively, using the same instrument so that results can be compared over time.

This report has 2 main limitations. First, molecular confirmation of Zika virus infection in the mother or the child was not possible. Second, we did not perform PCR or culture for cytomegalovirus. Nevertheless, the child’s Zika virus IgM was positive in the neonatal period, and her mother’s clinical symptoms suggested Zika virus infection during pregnancy, which occurred during the Zika virus epidemic in Brazil, in one of the most affected areas.

This case may indicate a broader spectrum in congenital Zika syndrome, raising questions about early prognostic markers. Our findings draw attention to the need for detailed evaluation even for typically developing children with possible congenital Zika virus infection who receive medical attention later.

Technical Appendix Additional information about infant with probable congenital Zika virus infection, Brazil.
