# Supplementary material for: Congenital Zika Virus Infection with Normal Neurodevelopmental Outcome, Brazil
Source: Emerg Infect Dis. 2018 Nov;24(11):2128–30. doi: 10.3201/eid2411.180883 (PMC6200011; doi:10.3201/eid2411.180883)
Supplement: Technical Appendix — Additional information about infant with probable congenital Zika virus infection, Brazil. [file 18-0883-Techapp-s1.pdf]

Article DOI: https://doi.org/10.3201/eid2411.180883

Congenital Zika Virus Infection with Normal Neurodevelopmental Outcome, Brazil

Technical Appendix

Results, Bayley-III Scales of Infant and Toddler Development

Cognitive subtest: Raw score of 56, Scaled score of 11, Composite score of 105.

Language subtest: Raw score of 20 for Receptive Language, Raw score of 21 for Expressive Language, Scaled score of 9 for both Receptive and Expressive Language, Composite score of 94.

Motor subtest: Raw score of 36 for Fine Motor, Raw score of 53 for Gross Motor, Scaled score of 11 for Fine Motor, Scaled score of 10 for Gross Motor, Composite score of 103.
